# Diabète en milieu urbain de Ouagadougou au Burkina Faso: profil épidémiologique et niveau de perception de la population adulte

**DOI:** 10.11604/pamj.2015.20.146.3249

**Published:** 2015-02-17

**Authors:** Georges Rosario Christian Millogo, Clément Yaméogo, André Samandoulougou, Nobila Valentin Yaméogo, Koudougou Jonas Kologo, Jean Yves Toguyeni, Patrice Zabsonré

**Affiliations:** 1 Service de Cardiologie du CHU Yalgado Ouédraogo, Ouagadougou, Burkina Faso

**Keywords:** Diabète, prévalence, glycémie, Diabetes, prevalence, glycemia

## Abstract

**Introduction:**

L'accroissement rapide de la prévalence du diabète sucré en Afrique subsaharienne constitue un problème de santé public. L'Organisation Mondiale de la Santé (OMS) estime qu’à l'horizon 2025, l'augmentation la plus significative de la prévalence du diabète sera enregistrée dans les pays en développement. Ceux-ci devraient abriter 75% des patients diabétiques du monde. Outre ses complications métaboliques, le diabète est un facteur de risque cardiovasculaire majeur. Le but de notre étude était de decrire le profil épidémiologique du diabète et d’évaluer le niveau de perception de la maladie par une population africaine en milieu urb qwqain de Ouagadougou.

**Méthodes:**

Le diabète a été défini chez tout sujet ayant une glycémie casuelle supérieure à 2 g/L ou une glycémie à jeun supérieur ou égale à 1,26 g/L (≥ 7 mmol/L) à deux controles d'une semaine d'intervalle. Etaient considérés diabétique, les sujets déjà suivis et ceux nouvellement dépistés par l'etude.

**Résultats:**

L’étude a inclus 1532 personnes, d’âge compris entre 25 et 64 ans dont 55,6% d'hommes et 44,4% de femmes. L’âge moyen était 36,10 ± 11,19 ans. La tranche d’âge de 25 à 34 ans était la plus représentée avec une proportion de 57,1%. La glycémie moyenne était de 1,04 g/L, la proportion des femmes ayant une hyperglycémie était statistiquement plus importante que celle des hommes (p< 0,05). Près de 81,3% de la population ignoraient que le diabète était un facteur de risque cardiovasculaire.

**Conclusion:**

Il est urgent de mettre en place un programme national de lutte contre le diabète sucré et les autres maladies non transmissibles au Burkina Faso.

## Introduction

L'accroissement rapide de la prévalence du diabète sucré en Afrique subsaharienne constitue un problème de santé public. L'OMS estime qu’à l'horizon 2025, l'augmentation la plus significative de la prévalence du diabète sera enregistrée dans les pays en développement, qui devraient abriter 75% des patients diabétiques du monde [1]. Le diabète est à l'origine de 60% des amputations des membres inférieurs dans ces pays [1]. Ces conditions appellent à la sensibilisation et à la participation de tous les acteurs intervenant dans la santé publique. Outre les maladies du pied, le diabète peut entraîner une cécité, des affections cardiaques et vasculaires et une insuffisance rénale. Au Burkina Faso, la prévalence du diabète est longtemps restée à l’état d'estimation en l'absence de statistiques fiables sur l'ampleur de la maladie dans la population générale. Ceci rend difficile les mesures de prévention. Le but de notre étude était de décrire le profil épidémiologique du diabète en zone urbaine africaine et d’évaluer le niveau de perception de cette population sur le diabète, afin d'envisager des mesures de sensibilisation et de prévention efficaces.

## Méthodes

Nous avons mené une étude transversale du 1^er^ au 31 Mai 2010 qui a recruté des hommes et femmes d’âge compris entre 25 et 64 ans dans la ville de Ouagadougou. Etait inclus dans l’étude les sujets appartenant à un ménage, habitant la ville depuis au moins un an. L'enquête s'est déroulée dans trois (03) arrondissements de la ville tirés au sort par échantillonnage aléatoire simple. La taille d’échantillon de chaque arrondissement était identique. Dans chaque arrondissement trois secteurs ont été tirés de façon aléatoire pour l'enquête et la taille d’échantillon allouée à chaque secteur était identique (soit 180 sujets par secteur). Les individus ont été recrutés dans les ménages et tous ceux qui répondaient aux critères d'inclusions ont été enquêtés. Pour le choix des ménages, nous avons numéroté les rues à partir de la plus grande voie du secteur, puis, une a été choisie de manière aléatoire pour le début de l'enquête. Les ménages situés de part et d'autre de la rue ont été enquêtés de proche en proche et lorsqu'on atteignait le bout de la rue sans que le nombre n'ait été atteint, la rue suivante à gauche était choisie jusqu’à ce que le nombre de sujets requis pour le secteur soit atteint. Le but et la démarche de notre enquête ont été expliqués aux sujets enquêtés après obtention préalable de leur consentement écrit ou verbal. Les outils ont été préalablement pretestés dans un autre secteur de la ville qui ne faisait pas partie de l'enquête finale. Un interrogatoire standardisé de 10 minutes a permis de recueillir les informations sur le sexe, l’âge, les antécédents personnels et familiaux de diabète, la date de la dernière glycémie et les connaissances sur le diabète. Le diabète a été définie chez tout sujet ayant une glycémie à jeun supérieur ou égale à 1,26g/L (≥ 7 mmol/L) ou glycémie casuelle supérieure à 2g/L après deux contrôles à une semaine d'intervalle [2]. La prévalence générale du diabète intégrait celle dépistée et les sujets ayant un antécédent personnel de diabète sucré. La glycémie a été mesurée à l'aide de lecteurs glycémiques avec des bandelettes réactives. Une goutte de sang capillaire d'environ un (01) millilitre a été prélevée à l'aide d'une lancette auto-piqueuse à usage unique chez les sujets à jeûn. L'Indice de Masse Corporelle (IMC) et le périmètre abdominal ont été utilisés pour apprécier l’état nutritionnel et anthropométrique des sujets. La définition de l'OMS a été utilisée pour évaluer le statut pondéral: obésité pour un IMC ≥30 kg/m^2^, surpoids pour un IMC compris entre 25-29,9 kg/m^2^ [3]. L'obésité abdominale a été définie selon la Nationnal Cholesterol Education Program-Adult Treatement Panel III (NCEP- ATP III) par un tour de taille ≥ 102 cm chez l'homme et ≥ 88 cm chez la femme [4]. L'IMC a été obtenu par le rapport du poids en kg sur la taille en m^2^. Le poids a été obtenu à l'aide des pèse-personnes gradués en Kg et le périmètre abdominal pris en position debout à l'aide d'un mètre ruban gradué en cm. La taille a été obtenue soit à partir de la carte d’état civil, soit mesurée à l'aide d'une toise graduée en cm. Les données validées ont été saisies sur micro-ordinateur et analysées à l'aide des logiciels EPI INFO Version 6.04 et SPSS Version 10.01 sous Windows. Les variables quantitatives continues ont été présentées sous forme de moyennes avec leurs intervalles de confiance à 95% et les variables qualitatives sous forme de proportions (%). Les tests d'inférence statistique ont été réalisés afin d'observer la distribution du phénomène dans la population de référence. Les tests de khi-deux de Mantel-Haenszel et les intervalles de confiance ont été utilisés. La valeur p

## Résultats

### Caractéristiques sociodémographiques

Sur 1600 personnes enquêtées, 1532 avaient des données complètes. Il y avait 852 (55,6%) hommes et 680 (44,4%) femmes, soit un sex-ratio de 1,25. La distribution de l’échantillon par classes d’âge et par sexe est représentée sur le Tableau 1. L’âge moyen de la population était de 36,10 ± 11,2 ans avec des extrêmes de 25 à 64 ans. La tranche d’âge de 25 à 34 ans était la plus représentée avec 57,1% des sujets. Les analphabètes étaient plus représentés (35,4%), suivis des sujets de niveau d'instruction secondaire (25%).

**Tableau 1:** Répartition de la population selon l’âge et le sexe

Classes d’âge (ans)	Sexe		Total n (%)
	Féminin n (%)	Masculin n (%)	
25-34	363 (41.5)	512 (58.5)	875 (57.1)
35-44	154 (47.5)	170 (52.5)	324 (21.1)
45-54	89 (50.0)	89 (50.0)	178 (11.6)
55-64	74 (47.7)	81(52.3)	155 (10.1)
Total	680 (100.0)	852 (100.0)	1532 (100.0)

### Caractéristiques du diabète dans la population

L'hyperglycémie a été diagnostiquée chez 130 personnes, soit une prévalence de 8,5% (IC_95%_= 7,2%; 10%). Les valeurs glycémiques variaient entre 0,4g/L et 3,40g/L. La glycémie moyenne était de 1,04g/L. La proportion des femmes ayant une hyperglycémie était significativement plus élevée que celle des hommes (10,4% vs 6,9%, p < 0,05). La prévalence de l'hyperglycémie augmentait très significativement avec l’âge. Le Tableau 2 indique la prévalence du diabète par tranche d’âge dans la population. Un antécédent familial de diabète est retrouvé chez 16,1% (n= 247) des personnes enquêtées. Les sujets de niveau socio-économique élevé étaient plus touchés par l'hyperglycémie (n= 32/236 soit 13,6%) que les autres sujets (98/1296 soit 7,6%; p= 0,0098). Dans la population enquêtée, 75,3% mesuraient pour la première fois la glycémie. Parmi les hyperglycémies diagnostiquées, 64,6% (n= 84) étaient des nouveaux cas dépistés au cours de l'enquête. Les anciens cas ont été dépistés lors d'une consultation ou d'une hospitalisation. En prenant en compte l'IMC, des sujets de notre échantillon, l'hyperglycémie était deux fois plus fréquente chez les sujets obèses ou en surpoids par rapport aux sujets de poids normal ou maigre (13,3% vs 5,9%, p < 0,00001) et était trois fois plus fréquente en cas d'obésité androïde (NCEP-ATP): 17,1% contre 5,9% chez le sujets ayant un périmètre abdominal normal. La différence était très significative (p < 0,0001).

**Tableau 2:** Prévalence de l'hyperglycémie et de l'obésité en fonction des classes d’âge

Classes âge (ans)	Hyperglycémie (p = 0,0000)	Obésité surpoids (p = 0,0001)	Obésité abdominale NCEP-ATP III (p < 0,0001)
25-34	38 (4,3)	232 (26,5)	119 (13,6)
35-44	33 (10,2)	152 (46,9)	107 (33,0)
45-54	17 (9,6)	85 (47,8)	64 (36,0)
55-64	42 (27,1)	64 (41,3)	61 (39,4)

### Niveau de connaissance de la population sur le diabète

Dans notre enquête, 96,3% de la population avaient déjà entendu le mot « diabète ». Ils étaient plus nombreux parmi les hommes et parmi les plus instruits. En revanche, seulement 287 sujets (19,7%) savaient que le diabète était un facteur de risque cardiovasculaire. Il n'existait pas de différence statistique entre le niveau de connaissance des hommes et celui des femmes et selon le niveau d'instruction (p= 0,0596). Le Tableau 3 résume les connaissances de la population sur le diabète en fonction du sexe et du niveau d'instruction.

**Tableau 3:** Connaissances de la population sur le diabète en fonction du sexe et du niveau d'instruction

connaissance sur le diabète		Sexe				Niveau d'instruction					
		**Homme**	**Femme**	**Les deux**	**p**	**Prim**	**Second**	**Sup**	**Postuni**	**Analph**	**p**
**Avoir entendu parler du diabète**	OUI	829 (97,3)	646 (95,0)	1475 (96,3)	0,0181	289 (97,3)	380 (97,4)	289 (97,4)	06 (100)	511 (94,1)	0,024
	NON	23 (2,7)	34 (5,0)	57 (3,7)		08 (2,7)	10 (2,6)	07 (2,4)	00 (0,0)	32 (5,9)	
**Diabète est un FRCV**	OUI	179 **(21,0)**	116 **(17,0)**	282 (19,3)	0,0596	56 (19,4)	71 (18,7)	67 (23,3)	03 (50,0)	90 (17,6)	0,1168
	NON	673 (79,0)	564 (82,9)	1237 (80,7)		233 (80,6)	309 (81,3)	222 (76,8)	03 (50,0)	421 (82,4)	

## Discussion

Les résultats de notre enquête montrent que l'implication du diabète dans la survenue des maladies cardiovasculaires est mal connue par la population urbaine même si cette population, dans sa grande majorité a déjà entendu parler de diabète. En effet l’étude montre que 93,6% de la population avait déjà entendu le mot diabète (communément appelé “*maladie du sucre*” en langue locale). Cependant, 80,3% de ces sujets ne considéraient pas le diabète comme étant un FRCV. Ni le sexe, ni le niveau d'instruction n'influençait sur les connaissances. En effet 82,9% des sujets analphabètes et 76,7% des sujets instruits pensaient que le diabète était une maladie à part entière sans aucune relation avec les maladies cardiovasculaires. La prévalence de l'hyperglycémie dans notre étude était de 8,5% dans la population. Faute de statistiques fiables, le diabète est longtemps resté à l’état d'estimation et de prévision. Dans les régions d'Afrique la prévalence se situe entre 3 et 20% selon les enquêtes STEP OMS [5]. Aujourd'hui l’étude menée à Ouagadougou révèle une situation inquiétante. Une étude hospitalière à Ouagadougou en 2010 sur un échantillon de 821 patients, rapportait une prévalence de 5,8% [6]. Cette forte prévalence a été observée dans plusieurs pays Africains en situation de transition épidémiologique. Kimbaly à Brazzaville [7], Yahia en Algérie [8] et Kane au Sénégal [9] trouvaient respectivement 6,8%, 7% et 10,5% en milieu urbain. Une grande variabilité existe avec les données de certaines études où des prévalences plus importantes ont été rapportées. Longo-Mbenza à Kinshasa trouvait une prévalence de 16% en milieu urbain [10], Zaoui à Tlemcen en Algérie, 15,3% en milieu urbain et 12,9% en milieu rural [11], Baldé en Guinée 15% en milieu urbain et 3% en milieu rural [12] tandis qu'elle était de 19,5% à l’île Maurice chez les sujets d’âge moyen. Selon l'OMS, si rien n'est fait, les prévalences actuelles pourraient augmenter de 98% entre 2010 et 2030 en Afrique noire [1]. Par ailleurs, notre étude indique une proportion significativement plus élevée chez les femmes (10,4% vs 6,9%, p < 0,001) et la prévalence du diabète augmentait avec l’âge et l'IMC. Il est clairement établi que l'obésité constitue un facteur de risque majeur dans la survenue du diabète sucré, surtout lorsqu'il existe en plus une obésité de type androïde [1, 11]. L'augmentation de la prévalence du diabète est manifestement liée à l'obésité, à la mauvaise alimentation et à la faible pratique d'activité physique sportive. Dans notre étude la prévalence du diabète était de 13,3% chez les sujets obèses ou en surpoids; 16% chez les obèses et 5,9% chez les sujets de poids normal ou maigre. En cas d'obésité abdominale associée la prévalence du diabète atteint 17,1%. Zabsonré, dans une étude hospitalière chez des patients obèses, retrouvait des résultats similaires avec une prévalence de 14% [13]. Le syndrome métabolique dont l'obésité centrale est une composante importante, favoriserait l'hyperglycémie et l'insulino-résistance rendant difficile la prise en charge du diabète déjà complexe et coûteuse dans un contexte de revenus limités. Ainsi, la surcharge pondérale est un facteur essentiel sur lequel pourrait se baser la politique de prévention primaire du diabète.

## Conclusion

Le diabète, facteur de risque cardiovasculaire majeur est très peu connu de la population urbaine quelque soit le niveau d'instruction et le sexe. Cette étude montre que l'hyperglycémie est fréquente dans la population burkinabè et que de nombreux cas sont méconnus. La prévalence se situe au même niveau que celle observée dans la plupart des pays en transition épidémiologique et les femmes semblent plus touchées par cette pandémie que les hommes. Cette étude fait également ressortir la nécessité de mettre en place un programme national intégré de lutte contre le diabète et des autres facteurs de risque cardiovasculaire associés par la collecte périodique des données, l'information et la sensibilisation de la population. La prise en charge très onéreuse et complexe du diabète et de ses complications dans les pays en développement, impose la prévention comme premier moyen de lutte.
